# Supplementary material for: MethylGenotyper: Accurate Estimation of SNP Genotypes and Genetic Relatedness from DNA Methylation Data
Source: Genomics Proteomics Bioinformatics. 2024 Jun 10;22(3):qzae044. doi: 10.1093/gpbjnl/qzae044 (PMC12016561; doi:10.1093/gpbjnl/qzae044)
Supplement: qzae044_Supplementary_Data [file qzae044_supplementary_data.zip › Figure S1.pdf]

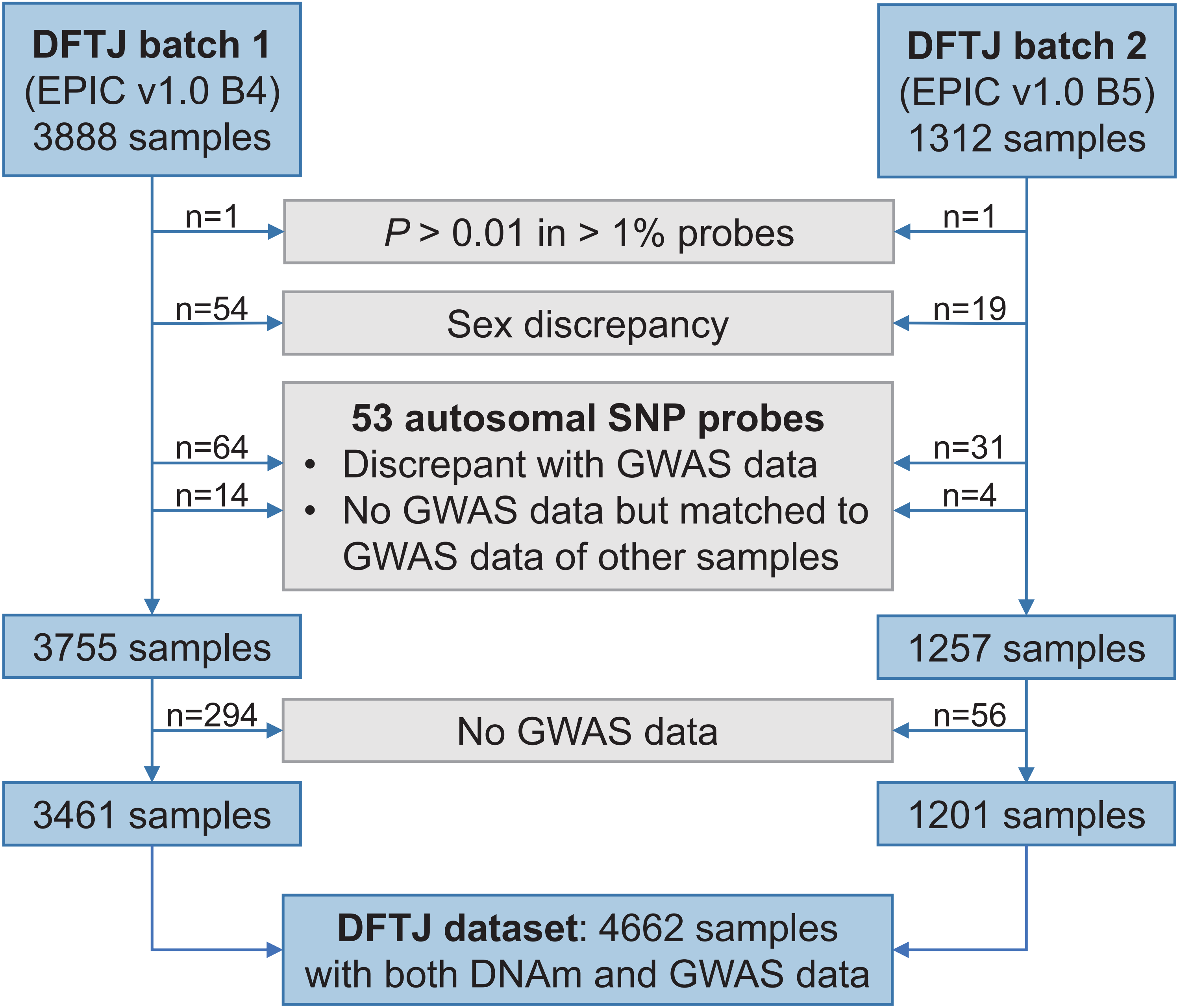
DFTJ batch 1
(EPIC v1.0 B4)
3888 samples
DFTJ batch 2
(EPIC v1.0 B5)
1312 samples
n=1
P > 0.01 in > 1% probes
n=1
n=54
Sex discrepancy
n=19
53 autosomal SNP probes
n=64
• Discrepant with GWAS data
n=31
n=14
• No GWAS data but matched to GWAS data of other samples
n=4
3755 samples
1257 samples
n=294
No GWAS data
n=56
3461 samples
1201 samples
DFTJ dataset: 4662 samples with both DNAm and GWAS data
