# Supplementary figures and images for: MethylGenotyper: Accurate Estimation of SNP Genotypes and Genetic Relatedness from DNA Methylation Data
Source: Genomics Proteomics Bioinformatics. 2024 Jun 10;22(3):qzae044. doi: 10.1093/gpbjnl/qzae044 (PMC12016561; doi:10.1093/gpbjnl/qzae044)

A

Density

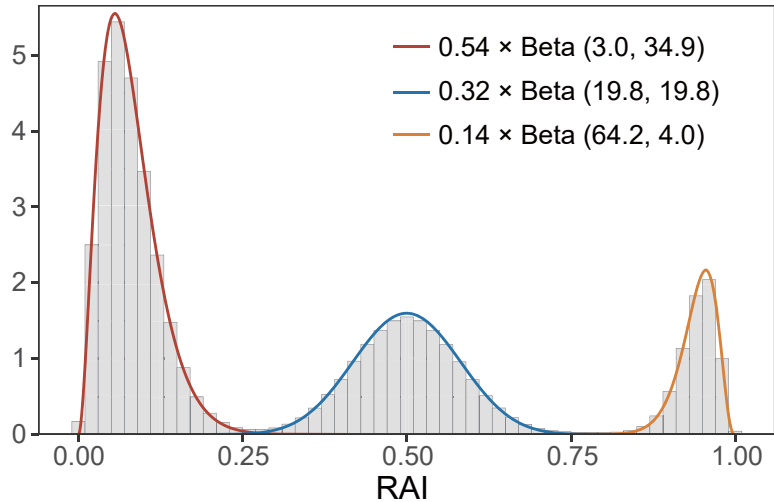

B

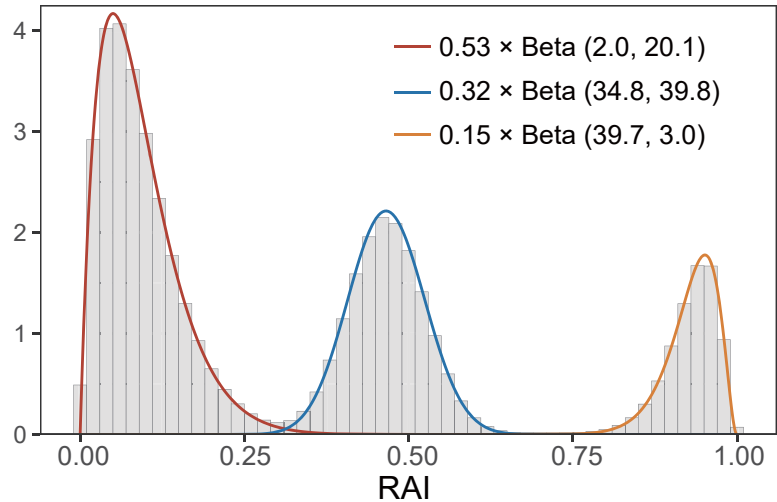

Supplement: qzae044_Supplementary_Data [file qzae044_supplementary_data.zip › Figure S3.pdf]

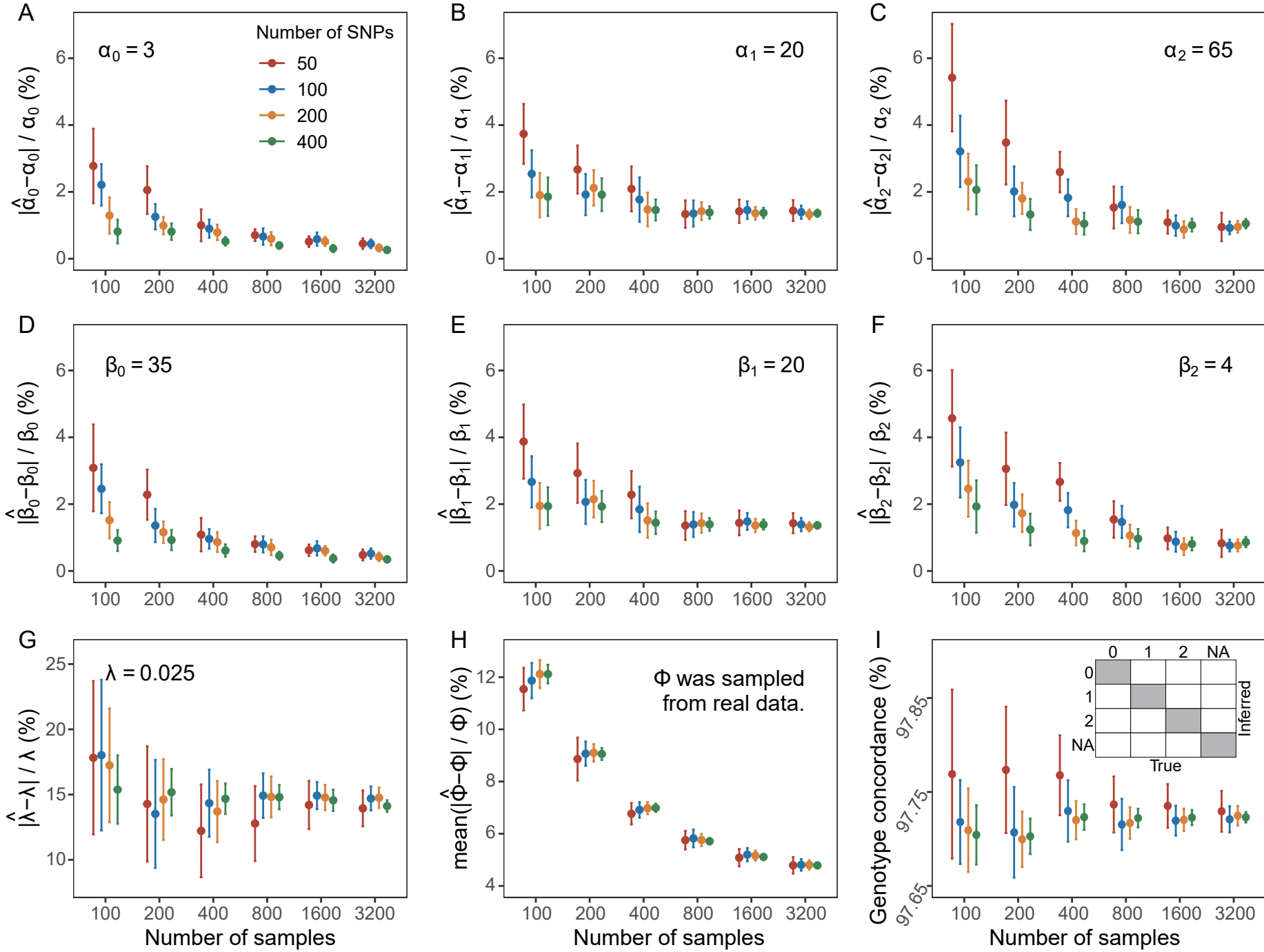

Supplement: qzae044_Supplementary_Data [file qzae044_supplementary_data.zip › Figure S4.pdf]

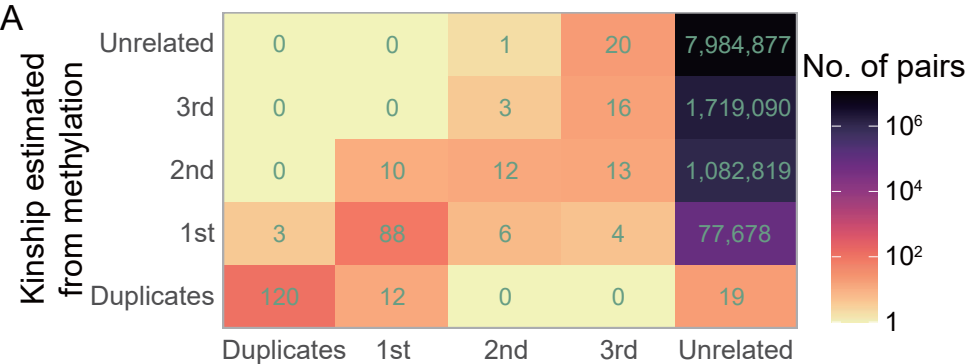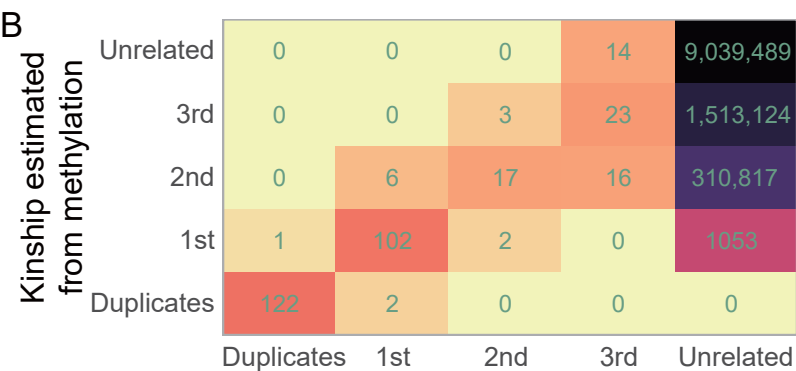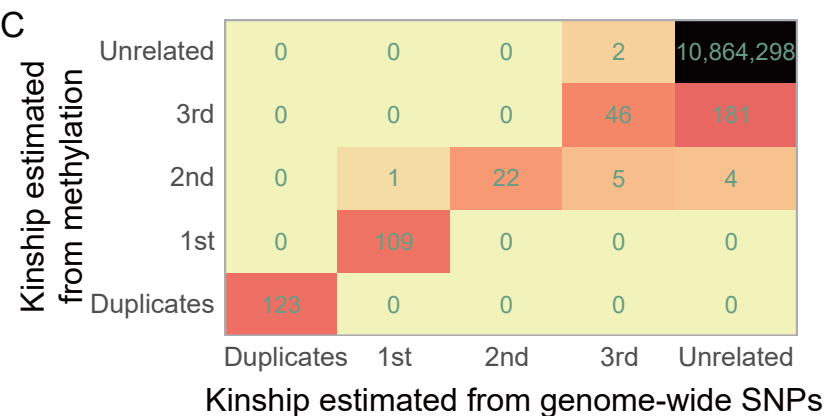

Supplement: qzae044_Supplementary_Data [file qzae044_supplementary_data.zip › Figure S5.pdf]

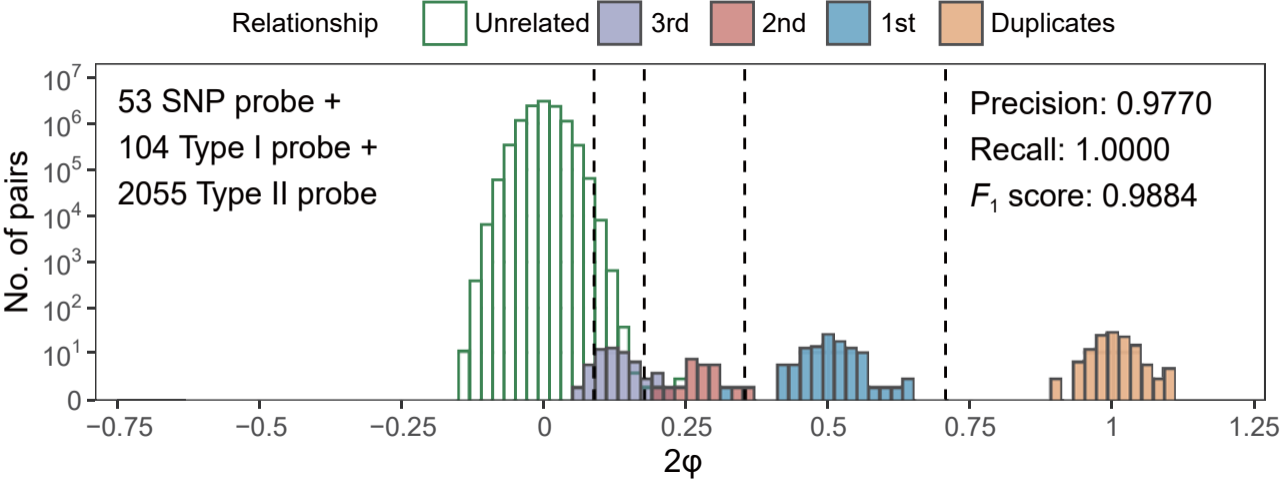

Supplement: qzae044_Supplementary_Data [file qzae044_supplementary_data.zip › Figure S6.pdf]

**A**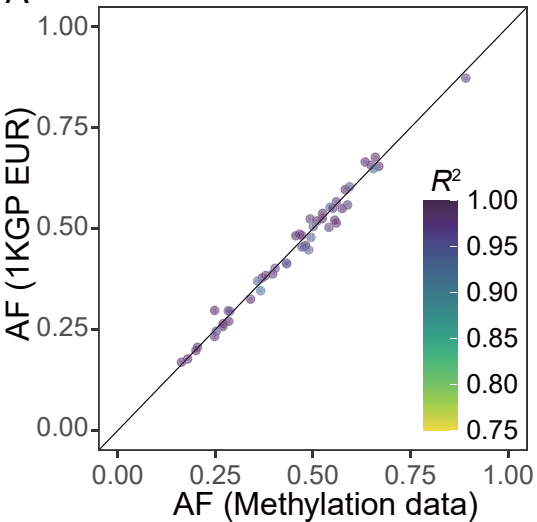**B**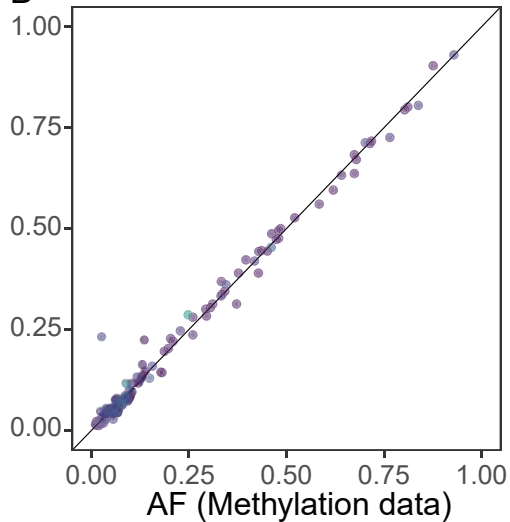**C**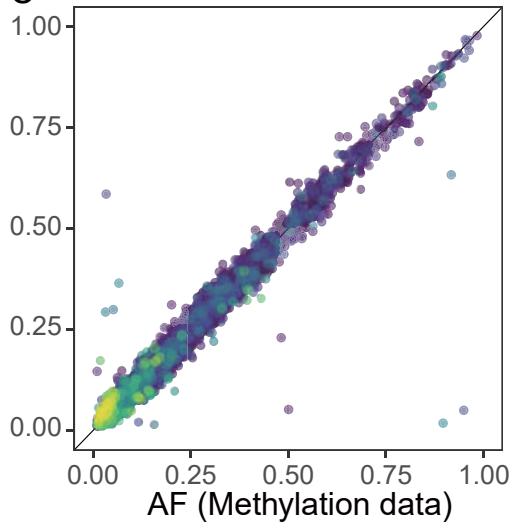

Supplement: qzae044_Supplementary_Data [file qzae044_supplementary_data.zip › Figure S7.pdf]
