# Supplementary material for: MethylGenotyper: Accurate Estimation of SNP Genotypes and Genetic Relatedness from DNA Methylation Data
Source: Genomics Proteomics Bioinformatics. 2024 Jun 10;22(3):qzae044. doi: 10.1093/gpbjnl/qzae044 (PMC12016561; doi:10.1093/gpbjnl/qzae044)
Supplement: qzae044_Supplementary_Data [file Figure S2.pdf › Figure S2.pdf]

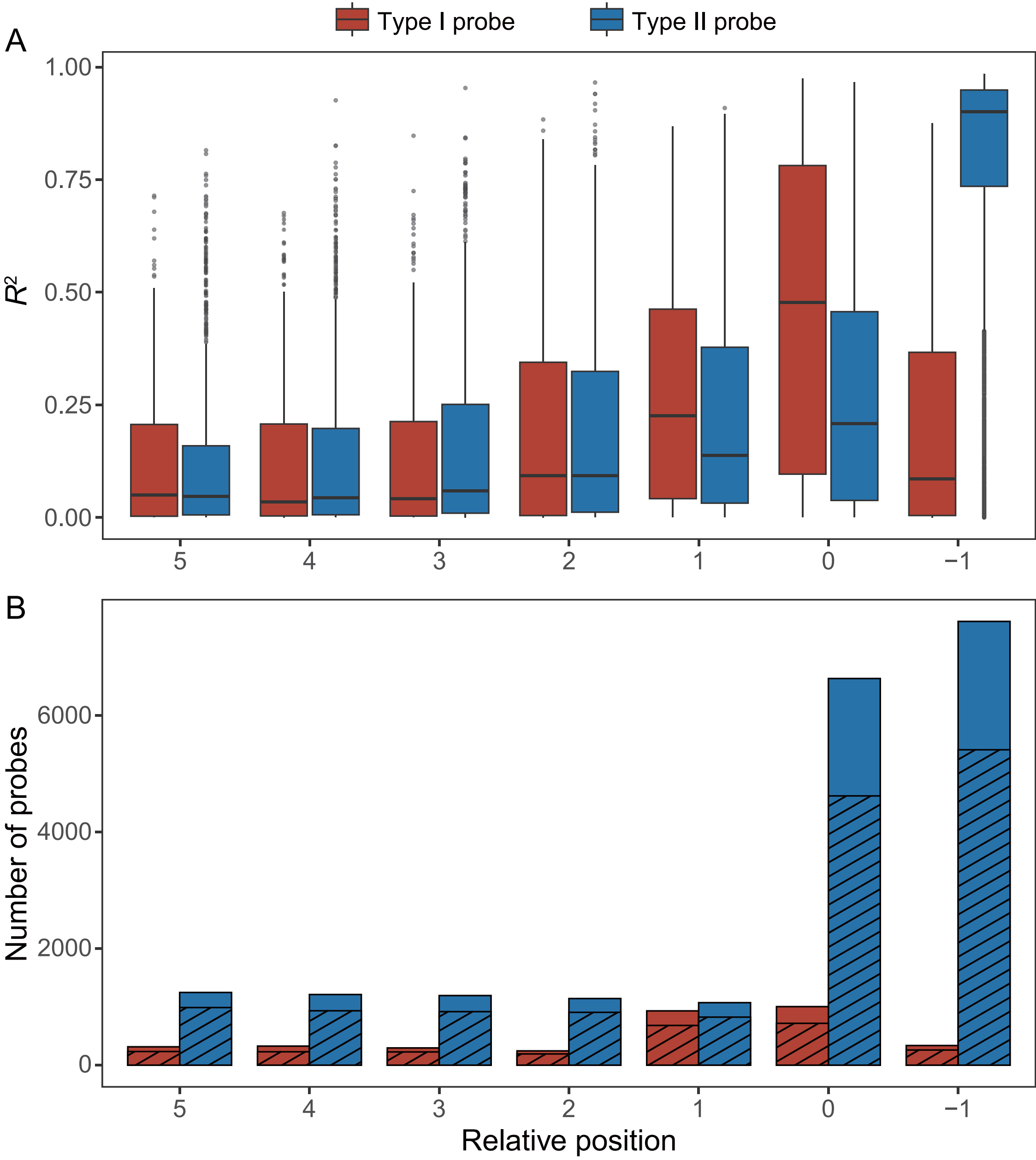

Type I probe
Type II probe
A
1.00
0.75
0.50
0.25
0.00
R^2
5
4
3
2
1
0
−1
B
6000
4000
2000
0
Number of probes
5
4
3
2
1
0
−1
Relative position
